# Some Observations Suggested by Mr. Cunningham's Case of Concussion of the Brain, Published in the February Number of the Medico-Chirurgical Journal

**Published:** 1818-07-01

**Authors:** A. P. Wilson Philip

**Affiliations:** Worcester


### VIII.

*Some Observations suggested by* Mr. Cunningham's *Case of Concussion of the Brain, published in the February Number of the Medico-Chirurgical Journal.*—By Dr. A. P. Wilson Philip, of Worcester.

To Dr. James Johnson.

Sir, *Worcester, April* 2, 1818.

I have endeavoured to point out, in my inquiry into the laws of the vital functions, that some of the experiments there related throw light on the nature of concussion of the brain, and the diagnosis between this disease and that produced by compression of this organ; and consequently on the diagnosis between nervous and sanguineous apoplexy. As an illustration of what I have said on this subject in the above inquiry, I beg to offer to your consideration the following observations on a well detailed case of concussion of the brain, by Mr. John Cunningham, published in the *Medico-Chirurgical Journal* for last February.

On examining the brain, after death, in this case, with the exception of "some turgescence" of the vessels of the dura and pia mater, the only morbid appearance found in the encephalon was, "that nearly about the middle of the right parietal bone there was in the cortical part of the cerebrum a blackish spot, about the size of a shilling, which appeared mashed or bruised, and its organization entirely destroyed." "The viscera of the chest and abdomen were perfectly sound." It appears, at first view, surprising, that a cause, producing so little apparent derangement, should excite so severe and rapidly fatal a train of symptoms. "Some turgescence" of the vessels of the dura and pia mater may certainly exist without producing such symptoms, and with regard to the mashed appearance of a small portion of the cortical part of the brain, we know that much larger portions of it may be wholly removed, without occasioning material injury to the vital functions. In many fatal cases of concussion, we find even less appearance of injury in the brain than in this case; and in some, little or none.

To what cause shall we ascribe the death of the patient in such cases? It is ascribed to the derangement of the finer mechanism of the brain, by which the functions of the nervous system are impeded; but the functions of the nervous system are also impeded in consequence of derangement of the finer mechanism of the brain by compression of this organ, yet the symptoms of concussion and compression are wholly different. The difference of the symptoms in these cases depends, I conceive, on the state of the sanguiferous system being different in them.

I have found, from repeated experiment, that the action of the heart is little influenced by uniform compression of the brain, but, that although the latter organ may be wholly removed without influencing the action of the heart, yet a powerful agent suddenly influencing it, affects the heart through it. Thus, if a ligature be thrown round the neck of an animal and the head be cut off, the heart will still be found beating with its usual force and regularity, and when the ligature is removed, will throw the blood from the arteries to a great distance; but if, instead of removing the brain, we crush it, the heart immediately loses its power. The ligature may be removed after the head has been cut off without the loss of more blood than what happens to be in the vessels.

I have repeatedly watched the action of the heart, after a severe blow had been inflicted on the brain. At first it flutters: in a short time it regains its regularity, except where the injury done is very extensive. Soon, however, it begins to beat more frequently and feebly, and often irregularly, and this state increases till the circulation fails altogether. In cases of compression, on the contrary, the power of the heart is great to the last, and the patient dies of suffocation, from the accumulation of phlegm in the lungs, the necessary consequence of failure of the nervous influence of the lungs, while the powers of circulation retain their vigour. In some cases, as must necessarily happen, the symptoms of compression and concussion are combined.

In concussion, the vessels of those parts of the brain which suffer from the blow are distended during the interval, in which considerable vigour of circulation prevails. In the above case, this stage had commenced before Mr. Cunningham had an opportunity of feeling the pulse. Had he examined it at the moment of the accident, he would have found it feeble and fluttering.

If the foregoing observations are correct, the principles of the treatment of compression and concussion are very different, if we except the removal of bones on other extraneous substances injuring the brain. In the former, the object is to relieve the distended vessels by lessening their contents, and reducing the vis a tergo. In the latter, we must obviate the tendency to distension, chiefly by lessening the contents of the vessels of the injured parts; we are assured that the disease itself will soon sufficiently reduce the vis a tergo; and as far as the tendency to distension in the head will admit of it, it is proper to support the vis a tergo. This, from the weakened state of the vessels of the brain, must be done with great caution in concussion, but in nervous apoplexy, which may be regarded as concussion, from internal causes, the cause of injury is not always of such a nature as necessarily to produce debility and consequent distention of the vessels of the head, and a more stimulating plan is often found beneficial. Compression is a disease of the brain alone: concussion, of both the brain and sanguiferous system.

I have addressed these observations to you, Sir, because I know the sanguiferous system has obtained much of your attention; and although many circumstances prevent my agreeing with you in your inferences from your ingenious experiment relating to that system, and I dare not flatter myself that my experiments will induce you to adopt the opinions to which I have been led, I am assured from your general character, that whatever is addressed to you will receive a candid hearing. If you consider the foregoing observations of sufficient importance to appear in the *Quarterly Register,* you will oblige me by inserting them.

I have the honour to be, Sir,

Your obedient humble Servant,

A. P. W. PHILIP.

